# Rupture of the Spleen, Discharge through Alimentary Canal, and Recovery

**Published:** 1871-05

**Authors:** Wm. Abram Love

**Affiliations:** Atlanta, Ga.


### *Rupture of the Spleen, Discharge through Alimentary Canal, and Recovery.* By Wm. Abram Love, M. D., Atlanta, Ga.

---

[*Read Before the Atlanta Academy of Medicine.*]

On the 29th December, 1863, I was called to see Tall, a servant of General Bryan, of Lee county. He was a dark mulatto, or copper-colored negro, perhaps one-eight Caucasian, six feet two or two and one-half inches in height, and, when in health, a well developed, muscular man, aged twenty-eight years.

He had suffered during the past season with repeated attacks of chill and fever, which had materially reduced his flesh and strength, and terminated in excessive enlargement of the spleen, causing it to extend far below the ribs, filling that portion of the abdominal cavity, and pressing the viscera adjacent into the left lumbar region.

In an altercation with a fellow servant, Tall received a severe kick, from a heavy brogan boot, striking directly over the centre of the enlarged spleen, from which he fell instantly, in a fainting condition.

A few hours after the injury, I found him lying on his left side, thighs flexed on abdomen, body bent forward, which position he assumed for the purpose of allaying the pain in the injured side. Reaction was not established, pulse feeble at 120, skin cold, as were the extremities, rigors, sick stomach and occasional vomiting—efforts to vomit producing great pain in the splenic region.

On examination, found a tumor or protrusion as large as a child's head, pressing against the inner wall of the abdomen, pushing the abdominal parietes outward from the region of the centre of the spleen. It was fluctuating, though tense, and tender under pressure. This condition, in my opinion at the time, was caused by extravasation of blood, in the trabecular tissue, from the breaking up of that substance in its abnormally softened condition, the result of the severe blow. Had the outer covering—the splenic capsule—been ruptured at the moment of receiving the injury, the case would have proved necessarily and speedily fatal, from loss of blood poured into the peritoneal cavity. Taking this view of the case, the prognosis was anything but encouraging, and the treatment was entered upon with little hope of doing more than giving temporary relief, until the capsule should give way, when hæmorrhage into the peritoneal cavity would soon terminate the case.

A bandage and compress was applied over the protuberance, opium given freely, with directions to repeat the dose when pain recurred, keeping him fully under its anodyne influence, and, whisky according to the indications, or the desire of the patient.

December 30.—Reaction taken place, with fever, pain still severe, protrusion more prominent, sickness at stomach diminished, though some nausea still at intervals, and seemingly not from the effects of the whisky nor opium. Bowels moved during the morning—discharge consisting of fæcal matter of ordinary consistence, presenting nothing unusual in appearance. As the following day was the regular time for a return of chill, and fearing that the additional pressure attendant upon the passive congestion of such a paroxysm would cause rupture, it was anticipated with full doses of Quinine, (grs. x, every two hours, until the system was brought under its influence) opium continued, milk punch, sinapisms and turpentine to spine.

December 31.—No chill, pain still severe, tumor more prominent, less resisting, involving apparently the entire spleen. Treatment continued, with some hope that adhesion would set up between splenic and abdominal parietal-peritoneal membranes, and give outlet to contents of capsule.

January 1.—Condition little changed, some fever; treatment continued.

January 3.—Fever continues, bowels have been evacuated, tumor protruding more pointedly; patient retains his flexed position, as one of most ease, and suffers much on moving. Continued treatment.

January 4.—On night of 4th, was summoned to see him in haste, with the report that he was suffering from cramp, and colic, and bleeding from the bowels. Reached him at 4 o'clock, A. M., of 5th (nearly seven days after injury). He had discharged from the bowels nearly *thirteen quarts* of bloody, grumous looking matter during the past four hours—each discharge being preceded by a gradually increasing pain, resembling colic, which was each time relieved by the evacuation. Skin and extremities cold; pulse thread-like, and barely perceptible, with every symptom of approaching dissolution. The tumor had entirely disappeared, the contents of the splenic capsule discharging through the alimentary canal. Milk punch and egg-nog were given freely; sinapisms to spine and extremities; per-salts of Iron, Quinine and Opium were administered liberally, alternated with Ergot and Tannic Acid, and strict quiet enjoined—a large compress and tight bandage having been applied. Again he discharged from the bowels a little more than *three* quarts of blood and matter, preceded, as before, by the "colic," after which he slept for several hours, during which time reaction commenced.

Within the ensuing twenty-four hours, he discharged eight quarts more of bloody matter, of about the same character and consistency, but colored, perhaps, by the Iron and Tannin. There was little or no coagulation. Treatment continued, leaving off the Tannin, and giving animal essence *ad libitum*, with punch of milk and eggs.

For the following five days, he discharged respectively—seven—five—three—two—and two quarts of bloody fluid, but little mixed with fæcal matter. After this time, the discharge diminished, and entirely disappeared by the end of another week.

During this period, the same plan of treatment was persisted in, leaving off the Ergot as the drain diminished.

The patient gradually recovered, regained his strength, and was in entire good health when last seen (in June, 1870), six years and a half after the injury.

I have never been able to trace the outline or identify any portion of spleen substance remaining, even with the most careful external manipulation. How much was entirely destroyed, or how much remains, I cannot say.

Some points of interest attach to this case, an allusion to which may not be out of place.

1st. That the case was one of rupture of the internal, or trabecular, substance of the spleen, without rupturing the splenic capsule, was evident from the sudden production of the tumor by the blow, and by the well defined circumscribed outline of the same.

2d. Had the capsule given way, death would have been inevitable and immediate, from hæmorrhage into the peritoneal cavity, or more remotely from extravasation into the cellular tissue.

3d. The contents of the splenic capsule must have been discharged into the left transverse arch of the colon, producing the "colic pain," and this perforation preceded by an adhesion between the splenic and colic peritoneum, either before or after the injury.

During six days, he discharged over *forty quarts* of blood, blood-fluid, broken-down trabecular tissue, &c., from the splenic suppuration. This is, of course, inclusive of fæcal matter and alimentary excreta, which, perhaps, did not, in all, exceed six or eight quarts.

Rupture of the spleen is so certainly fatal in its termination, that this case is regarded as not without interest. Though not one of immediate rupture of the capsule, it was of the trabecular tissue, and from the short time elapsing before the giving way of the covering, it may fairly be classed as a case of *rupture* of the spleen.

What influence the alternation of the Ergot and per-salts of Iron, (*per sulphate and per chloride,*) may have over the blood and the bleeding vessels under such circumstances, is left for others to determine for themselves. As for myself, I have so long used them to fill the therapeutic indications, as I understand them, and with so much satisfaction as to results, that I am inclined to cling to both—their alternate use.

The history of this case very naturally gives rise to the query: May not many of the cases of fatal hæmorrhage from the bowels, in cases of excessive enlargement of the spleen, with softening of its trabecular substance, be the result of rupture through the adherent colon? Such a result, following the super-engorgement of the softened splenic vessels, induced by the chill paroxysm, may not be impossible. The speedily fatal termination of a few cases, under such apparent circumstances, when the attendant symptoms were markedly allied to those given in this case, has induced this supposition, since witnessing the above case. In those referred to, there existed no evidence of hæmorrhagic cachexia, or predisposition to, or premonitions of, such speedily fatal termination, until the bloody, grumous looking fluid was poured in profusion from the bowels, from which the patients soon perished. An autopsy may have revealed an opening between the colic canal and the internal splenic tissue, in its softened condition, but the popular prejudice against post mortem examinations in this country would not admit of an autopsy.

In conclusion, I will take this occasion to say that, in my clinical observation, I have not found the *full-blood* African subject to enlargement of the spleen from malarial influences, nor is the per cent. of fatality from visceral hæmorrhage so great in the black, as in the white race, in the malarial regions of this country.

*Junction of Peachtree and Broad Streets*, April 28, 1871.

